# Association between Bathing and Survival in Patients with Advanced Cancer in Their Last Days of Life: A Prospective Cohort Study

**DOI:** 10.1089/pmr.2020.0111

**Published:** 2021-03-12

**Authors:** Kiyofumi Oya, Tatsuya Morita, Hidenobu Koga, Masanori Mori, Hideyuki Kashiwagi, Takashi Ohmori, Yaichiro Matsumoto, Eri Matsumoto, Shunsuke Kosugi, Sho Sasaki

**Affiliations:** ^1^ Department of Transitional and Palliative Care, Iizuka Hospital, Fukuoka, Japan.; ^2^ Palliative and Supportive Care, Seirei Mikatahara General Hospital, Hamamatsu City, Shizuoka, Japan.; ^3^ Clinical Research Support Office, Iizuka Hospital, Fukuoka, Japan.; ^4^ Department of General Internal Medicine, Iizuka Hospital, Fukuoka, Japan.; ^5^ Department of Nephrology, Iizuka Hospital, Fukuoka, Japan.; ^6^ Department of Healthcare Epidemiology, Kyoto University Graduate School of Public Health, Kyoto, Japan.

**Keywords:** bathing, end-of-life care, neoplasm, nursing care, palliative care

## Abstract

***Background:*** Although many Japanese patients wish to take a bath in their last days, the safety of bathing for patients with a prognosis of a few days is not known.

***Objective:*** To examine whether taking a bath affects the survival of advanced cancer patients with prognoses of a few days.

***Design:*** A single-center prospective cohort study.

***Setting/Subject:*** Advanced cancer patients in their last days of life in a palliative care unit of a Japanese hospital. We compared patients who took baths with those who did not. The primary endpoint was 24-hour survival rate.

***Result:*** Among 110 patients eligible for this prospective study, 89 (72%) met the inclusion criteria. Forty-eight patients (43%, 223 person-days) were eligible for analysis. A total of 28 patient-days were classified into the bathing group, and 192 patient-days were classified into the nonbathing group. After propensity score matching, the 24-hour death rate was 10.7% in the bathing group and 8.0% in the nonbathing group, respectively (mean difference 2.8% with 95% confidence interval of −11.2% to 16.8%, *p* = 0.65).

***Conclusion:*** Taking a bath does not appear to bear a significant association with shortening of life among advanced cancer patients in their last days of life.

## Introduction

Optimal care for terminally ill patients should be individualized to reflect each patient's preferences, hopes, and values.^1^ Because patient preferences for care are shaped by the patient's sociocultural context,^2^ it is essential to consider the cultural background of patients.

One of the unique cultural aspects of Japan is bathing. Most Japanese people love deep hot tub bathing (o-furo) rather than showers or saunas.^3^ Public opinion surveys have demonstrated that >90% of Japanese people show a preference for taking baths.^4^ The average duration of a bath is 25.2 minutes and ∼80% to 95% of people take a tub bath every day.^4^ Bathing is seen as an essential daily behavior not only to keep the body clean but also to relieve fatigue, refresh the body and mind, and to improve general well-being.^5^ Historically, bathing has been a religious way of removing defilements.^6^ Therapeutic bathing also contributed to the origin of bathing.^6^

A considerable number of patients want to take a bath even in their last days.^7^ In recent studies, hot tub bathing was shown to reduce pain/anxiety, induce relaxation without affecting vital signs, and improve well-being among advanced cancer patients.^8,9^ To provide assisted tub bathing, at least two professional caregivers are necessary. Despite the need for human resources, a recent Japanese inpatient hospice care survey demonstrated that 40% of terminally ill cancer patients received assisted tub bathing.^10^ In a nationwide survey of bereaved families, >90% of families believed that caregivers should provide what patients want for comfort, such as baths and massages.^11^ The issue of whether patients can safely take baths in their last days of life is, therefore, relevant not only to patients but also to their families.

The safety of bathing for patients with a prognosis of a few days is not known. To our knowledge, no study of bathing has evaluated the survival of advanced cancer patients. Because of its potential for harm, nurses sometimes hesitate to allow tub bathing for patients with a few days to live.^12^

The objective of this study was to examine whether taking a bath affects the survival of advanced cancer patients with prognoses of a few days. We hypothesized that there is no association between survival and taking tub baths.

## Methods

### Study design and participants

This study was a single-center prospective cohort study of advanced cancer patients with prognoses of a few days. We compared patients who took baths with those who did not.

The Institutional Review Board of our institution approved the scientific and ethical validity of this study, which follows Declaration of Helsinki principles and the Strengthening the Reporting of Observational Studies in Epidemiology guidelines for reporting.^13^

We planned consecutive enrollment of terminally ill cancer patients who were admitted to the palliative care unit at Aso Iizuka Hospital from January 1, 2017 to March 31, 2018, or until the total number of patients reached 110. The inclusion criteria were age ≥18 years and a palliative performance score (PPS) of ≤20, which connote a median survival of two to six days.^14–16^

### Procedure

The data were prospectively recorded by the patients' primary physicians. We collected the baseline characteristics on admission. After the PPS dropped to <20 longitudinal data were recorded daily. We followed the patients for 14 days or patient death, whichever came first.

The decision as to when a patient would be bathed was made in discussion between the nurses and the patient, based on the patient's wishes and the resources in the facility. The assisted bathing setup that we used is commonly available in many Japanese hospices and palliative care units. In Japanese-style tub bathing, after washing the body and the hair with assistance from nurses using a shower head or other device, patients soak in a hot tub up to their shoulders. The bathing hot water temperature was adjusted to ∼41°C. The length of time in the bath was not fixed, and patients and nurses decided when to leave the tub by mutual agreement.

### Measurement

We collected patient gender, age, primary cancer site for baseline characteristics. For longitudinal data, we monitored the number of clinical signs of impending death^17^ (which consisted of decreased response to verbal stimuli, decreased response to visual stimuli, drooping of nasolabial folds, hyperextension of neck, grunting respirations, dysphagia for liquids, apnea, Cheyne–Stokes respiration, mandibular [open-mouth] breathing, peripheral cyanosis, pulselessness of the radial artery, inability to close the eyes, and decreased urine output); three vital sign changes (i.e., body temperature ≥38°C; respiratory rate ≥20/minute; systolic blood pressure, ≤90 mmHg); need for oxygen inhalation^18^; and tub bath events.

### Primary endpoint

The primary endpoint was the 24-hour survival rate. We measured the primary endpoint by chart review. The reason for this choice was that the bathing effect was presumed to last for several hours at most.^19^ We did not use time-to-event methods (e.g., Kaplan–Meier survival curves and Cox proportional hazards models) because some patients experienced several tub baths in the time period. Instead, based on the assumption that the baseline risk of death was equivalent all the time, we used patient-day data for this study.

### Statistical analysis

For analyses, we excluded 15 patients who died within one day after inclusion because we assumed that such actively dying patients had no chance to take a bath and could be excluded from the patient population. We also excluded 16 patients who survived ≥15 days after the PPS dropped to <20 because the score was inaccurate with regard to prognosis. As we used person-time data (i.e., patient-day data), we defined the bathing group as patient-days who experienced tub bathing, and the nonbathing group as patient-days who did not. We used the propensity score kernel matching method^20^ to compare the bathing group with the nonbathing group. Regarding covariates, we used the number of clinical signs of impending death, the three vital signs, oxygen inhalation, and number of days after a PPS ≤20. In this matching, both groups were matched within 20% of the caliper of the standard deviation of the propensity score. We considered a standardized mean difference <10% as indicative of good covariate balance.^21,22^ We estimated the average treatment effect on treated because not all patients wanted to take baths, and we wanted to discover the magnitude of the bathing effect among those who wished to take baths. We assumed the missing data were ignorable if the proportion was <5% in each covariate. We used Stata/SE ver.14.2 for the data analysis. A two-sided *p* < 0.05 was considered to be statistically significant.

## Results

### Patient characteristics

**FIG. 1.**
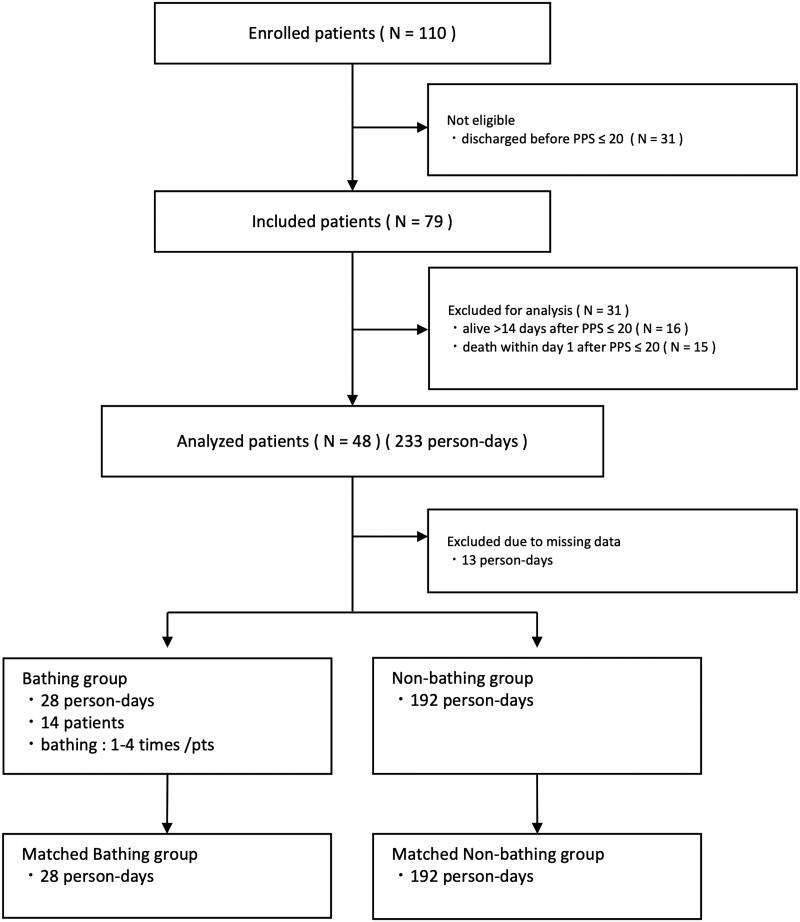
Flow diagram of this study.

Figure 1 shows the flow diagram of this study. Among 110 patients eligible for this prospective study, 89 (72%) met the inclusion criteria. A total of 31 patients (28%) were excluded due to discharge from the palliative care unit before their PPS was ≤20. For analysis, we further excluded patients who were still alive >14 days after their PPS was ≤20 (16 patients, 15%), and those who died within one day after the PPS dropped to ≤20. Finally, 48 patients (43%, 223 person-days) were eligible for analysis. Table 1 summarizes the patient's characteristics. Fourteen patients took baths at least once. A total of 28 patient-days were classified into the bathing group, whereas 192 patient-days were classified into the nonbathing group.

**Table 1.** Patient Characteristics

	Patients (*n* = 48)
Age	
Mean (SD)	72.3 (11.7)
Median (IQR)	72.5 (66–80.5)
Gender	
Male	32 (66.7%)
Female	16 (33.3%)
Primary cancer site	
Lung	9 (18.8%)
Stomach	6 (12.5%)
Colon/rectum	5 (10.4%)
Liver	4 (8.3%)
Pancreas/bile ducts	8 (16.6%)
Ovary/uterus	3 (6.3%)
Prostate	3 (6.3%)
Head and neck/brain	3 (6.3%)
Unknown	1 (2.1%)
Others	6 (12.5%)
Tub bath experience	
None	31 (64.6%)
Once	8 (16.7%)
Twice	8 (16.7%)
Three times	0 (0%)
Four times	1 (2.1%)

Data are *n* (%) unless otherwise specified. All characteristics were recorded at enrollment, apart from tub bath experience.

### Propensity score matching balance

Table 2 shows the covariance balance. After kernel matching, the covariates were well balanced across bathing and nonbathing groups, within 10% of the standardized mean difference.

**Table 2.** Signs of Impending Death before and after Propensity Score Matching

Covariates	Unmatched			Matched		
	Bathing	Nonbathing	SMD	Bathing	Nonbathing	SMD
	(*n* = 28)	(*n* = 192)	(%)	(*n* = 28)	(*n* = 192)	(%)
Clinical signs of impending death (no.)^a^	0.76	1.8	−62.2	0.79	0.85	−3.8
Body temperature ≥38.0°C (%)	0.69	0.14	−22.4	0.07	0.09	−4.7
Abnormal respiratory rate (%)^b^	0.36	0.38	−4.8	0.36	0.33	4.9
Systolic blood pressure ≤90 mmHg (%)	0.1	0.21	−29.9	0.11	0.13	−7.3
Oxygen inhalation (%)	0.34	0.66	−66.5	0.32	0.31	1.7
Day after PPS ≤20 (no.)	3.9	4.62	−21.6	3.7	3.8	−2.1

^a^ This included the following clinical signs: decreased response to verbal stimuli, decreased response to visual stimuli, nasolabial fold drooping, neck hyperextension, grunting respirations, dysphagia for liquids, apnea, Cheyne–Stokes respiration, mandibular breathing, peripheral cyanosis, pulselessness of the radial artery, inability to close the eyes, and decreased urine output.

^b^ Respiratory rate <10/minute or >20/minute.

PPS, palliative performance score; SMD, standardized mean difference.

### Bathing and survival rate

Table 3 shows the 24-hour survival rate before and after propensity score matching. In the unmatched cohort, the 24-hour death rate was 10.7% in the bathing group and 16.1% in the nonbathing group, respectively. After propensity score matching, the 24-hour death rate was 10.7% in the bathing group and 8.0% in the nonbathing group, respectively (mean difference 2.8% with 95% confidence interval of −11.2% to 16.8%, *p* = 0.65).

**Table 3.** Twenty-Four-Hour Survival Rate before and after Propensity Score Matching

	Bathing (n = 28)	Nonbathing (n = 192)	Mean difference	95% Confidence interval	*p*
Unmatched 24-hour survival rate (%)	10.7	16.1	5.4	(−20.0 to 9.2)	0.23
Matched 24-hour survival rate (%)	10.7	8.0	2.8	−11.2 to 16.8	0.65

## Discussion

To our knowledge, this is the first study to explore the association between bathing and survival among patients with prognoses of a few days. The main finding is that taking a tub bath was not associated with a shortening of life among patients with prognoses of several days.

Some doubt the relevance of the 24-hour survival of actively dying patients because their interest is not always in sustaining their lives but in how comfortable they can be in the last days of life. However, from the perspective of families and health care providers, this may be a highly important issue because any treatments or nursing activities that potentially affect patient survival can cause a negative focus on emotional or ethical issues. If a patient dies soon after bathing, caregivers may associate one with the other and feel guilt, which potentially leads to complicated grief^23^ or burnout.^24^ Our findings suggesting that tub baths are not associated with a shortening of survival might reassure families and health care providers.

This study has considerable limitations. First, this study was a single institute study. It is thus difficult to generalize the study result with other settings. Second, we did not measure how many patients actually wanted to take baths across the study period. The sample size of this study was also small. Further large studies are warranted to confirm the results. Third, there was a large difference in the number of patients between the two groups. Fourth, there may have been unmeasured confounding factors such as consciousness level or acute complications (e.g., acute pneumonia and gastrointestinal hemorrhage). Propensity scores only balance measured covariates, a situation that does not necessarily indicate balance in unmeasured covariates. We do not believe, however, that this seriously influenced the conclusion because we selected major confounding factors that were relevant to the causal relationship.

## Conclusion

Taking a bath does not seem to bear a significant association with shortening of life among advanced cancer patients in their last days of life. This result might lessen the emotional burden of professional caregivers and families when a patient wants to take a bath. This study may aid in the design of a further prospective cohort study, as a larger study is needed to confirm these results.

## Abbreviations Used

PPS: palliative performance score
SMD: standardized mean difference
